# The relationship of self-efficacy to catastrophizing and depressive symptoms in community-dwelling older adults with chronic pain: A moderated mediation model

**DOI:** 10.1371/journal.pone.0203964

**Published:** 2018-09-18

**Authors:** Sheung-Tak Cheng, Candi M. C. Leung, Ka Long Chan, Phoon Ping Chen, Yu Fat Chow, Joanne W. Y. Chung, Alexander C. B. Law, Jenny S. W. Lee, Edward M. F. Leung, Cindy W. C. Tam

**Affiliations:** 1 Department of Health and Physical Education, The Education University of Hong Kong, Hong Kong SAR, China; 2 Department of Clinical Psychology, Norwich Medical School, University of East Anglia, United Kingdom; 3 Department of Anaesthesiology & Operating Services, Alice Ho Miu Ling Nethersole Hospital, Hong Kong SAR, China; 4 Department of Anaesthesiology & Operating Theatre Services, Queen Elizabeth Hospital, Hong Kong SAR, China; 5 Department of Medicine and Geriatrics, Princess Margaret Hospital, Hong Kong SAR, China; 6 Department of Medicine, Alice Ho Miu Ling Nethersole Hospital, Hong Kong SAR, China; 7 Department of Medicine and Geriatrics, United Christian Hospital, Hong Kong SAR, China; 8 Department of Psychiatry, North District Hospital, Hong Kong SAR, China; University of Malaya, MALAYSIA

## Abstract

Self-efficacy has been consistently found to be a protective factor against psychological distress and disorders in the literature. However, little research is done on the moderating effect of self-efficacy on depressive symptoms in the context of chronic pain. This cross-sectional study aimed to examine if pain self-efficacy attenuated the direct relationship between pain intensity and depressive symptoms, as well as their indirect relationship through reducing the extent of catastrophizing when feeling pain (moderated mediation). 664 community-dwelling Chinese older adults aged 60–95 years who reported chronic pain for at least three months were recruited from social centers. They completed a battery of questionnaires on chronic pain, pain self-efficacy, catastrophizing, and depressive symptoms in individual face-to-face interviews. Controlling for age, gender, education, self-rated health, number of chronic diseases, pain disability, and pain self-efficacy, pain catastrophizing was found to partially mediate the connection between pain intensity and depressive symptoms. Furthermore, the relationship between pain intensity and depressive symptoms was moderated by pain self-efficacy. Self-efficacy was also found to moderate the relationship between pain intensity and catastrophizing and the moderated mediation effect was confirmed using bootstrap analysis. The results suggested that with increasing levels of self-efficacy, pain intensity’s direct effect on depressive symptoms and its indirect effect on depressive symptoms via catastrophizing were both reduced in a dose-dependent manner. Our findings suggest that pain self-efficacy is a significant protective factor that contributes to psychological resilience in chronic pain patients by attenuating the relationship of pain intensity to both catastrophizing and depressive symptoms.

## Introduction

Chronic pain can be a debilitating condition and is common among older adults. Some estimates suggest that half or more of community-dwelling older adults and up to 80% of nursing home residents suffer from chronic pain [1]. Arthritis (e.g., rheumatoid arthritis, osteoarthritis), back pain, and fibromyalgia are the most common pain disorders in older people [2,3]. Injuries (e.g., falls) and surgical procedures also contribute to the higher rates of pain in older adults. People with chronic pain, regardless of age, suffer from impaired functioning, reduced quality of life, and depression [4–6]. More research is needed to understand pain, especially factors that can alleviate pain and its impacts, in older adults in light of the rapidly increasing number of older pain patients due to global aging. In this study, we articulate a new cognitive model of pain and depression and tested it in a community sample of Chinese older adults in Hong Kong, although the model may be applicable to younger persons with chronic pain as well. In this model, self-efficacy and catastrophizing play a moderational and a mediational role respectively in the connection between pain and depression. What distinguishes this model from the others in the existing literature is how these factors are put together in an integrated model.

Some scholars have suggested that catastrophizing, a tendency to exaggerate pain sensation, to ruminate about it, and to feel helpless because of it [7], would mediate the relationship between pain and depressive symptoms. In a cross-sectional study of 164 back pain patients, Hülsebusch and colleagues found that the effect of pain intensity was completely mediated by pain-related cognitions, with help-/hopelessness being the most important pathway leading to depressive symptoms [8]. Wood and colleagues measured pain intensity, depressive symptoms, and catastrophic cognitions (namely, magnification and helplessness) at baseline and six months later in 141 older adults with chronic non-cancer pain. Using change scores from baseline to follow-up, they found that both magnification and helplessness completely mediated the connection between pain intensity and depressive symptoms [9].

Whereas catastrophizing is a risk factor for depression, self-efficacy, in terms of believing in one’s ability to carry out necessary actions to manage pain and to reduce the impact of pain on everyday activities [10,11], appears to have the reverse effect. Studies have found that patients with comparable levels of pain tend to have less depressive symptoms if they have higher pain self-efficacy [12,13]. In the extant literature, self-efficacy is usually treated as a mediator between pain and depressive symptoms [14,15]. Such a conceptual model assumes (the lack of) self-efficacy to arise from pain. This is possible as severe pain may undermine one’s sense of self-efficacy. However, self-efficacy is a psychological resource that may serve as a protective factor against pain-induced depression; in this sense, self-efficacy would moderate the relationship between pain and depressive symptoms.

To the best of our knowledge, Pjanic and colleagues were the only ones that looked at the moderating role of self-efficacy. They followed 274 patients suffering from accidental injuries for 12 months and found that baseline self-efficacy moderated the effect of pain intensity and impairment at baseline on depressive symptoms at follow up, controlling for depressive symptoms at baseline. Pain strongly predicted depressive symptoms when self-efficacy was low, but was basically unrelated to depressive symptoms when self-efficacy was high [16]. In other words, the effect of pain on depression depends on the level of self-efficacy.

**Fig 1:**
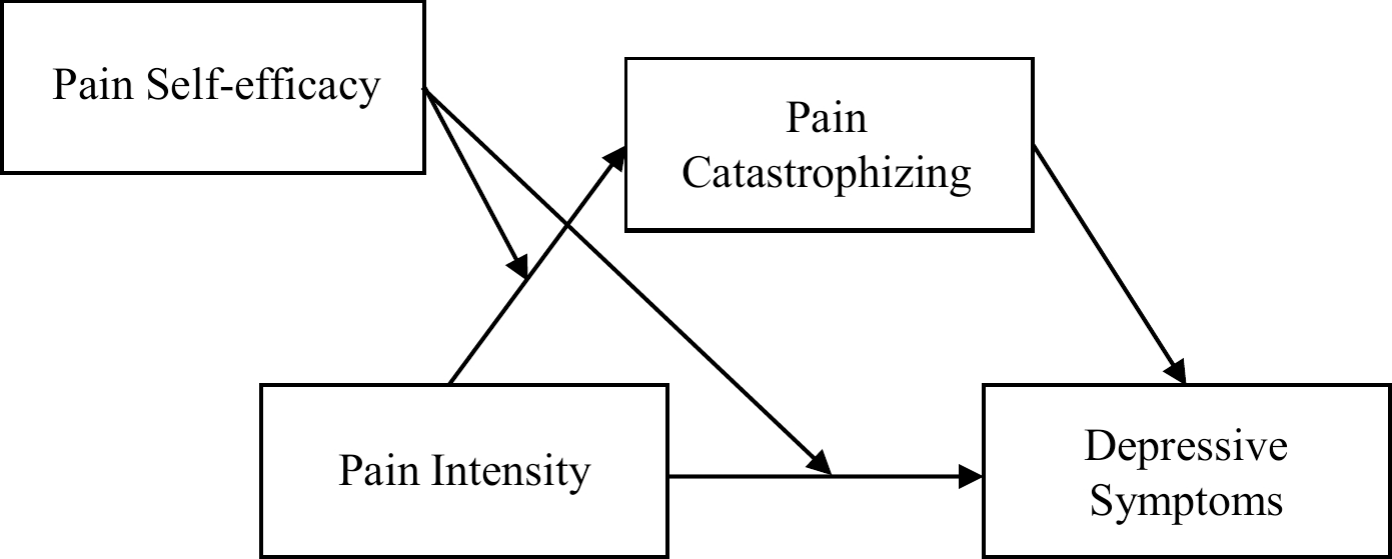
Hypothesized model of the interrelationships among pain intensity, pain catastrophizing, pain self-efficacy, and depressive symptoms.

However, Pjanic and colleagues [16] did not include catastrophic cognitions in their study. This study proposes a new, moderated mediation model (Fig 1) that incorporates the role of catastrophizing and tested the model in a sample of community-dwelling older adults in Hong Kong. In addition to acting on the relationship between pain and depressive symptoms, self-efficacy is hypothesized to moderate the association between pain and catastrophizing, while catastrophizing is in turn a precursor of depressive symptoms (Fig 1). In other words, pain intensity is postulated to have stronger relationships with *both* catastrophizing and depressive symptoms when self-efficacy was low than when self-efficacy was high, while catastrophizing also serves as a mediator for the connection between pain intensity and depressive symptoms. Thus, pain intensity is hypothesized to increase depressive symptoms both directly when self-efficacy was low, as well as indirectly through increasing catastrophizing when self-efficacy is low.

## Methods

### Study design, participants and procedure

Between September, 2015 and March, 2016, 941 older adults were recruited on a voluntary basis from social centers for older people in Hong Kong to participate in a cross-sectional survey about chronic pain. They provided written informed consent to participate and were interviewed face-to-face individually by trained research assistants in a quiet area of the social center. 664 persons who met the inclusion/exclusion criteria were included in this study. The inclusion criteria were (1) aged ≥60 years, (2) a native Chinese speaker, and (3) duration of pain ≥3 months. The exclusion criterion was presence of cognitive impairment being operationalized as a score of ≥3 on the Short Portable Mental Status Questionnaire [17]. The sample (*N* = 664) had a mean age of 74.79 years (*SD* = 7.20; range = 60–95), with majority of them being women (85.1%). About half of the participants were married or cohabiting (49.3%), while 43.8% of them were widowed. Over 70% of them received formal education (primary = 43.7%; secondary = 23.6%; tertiary = 4.1%). The study was approved by the Human Research Ethics Committee of The Education University of Hong Kong.

### Measures

#### Depressive symptoms

The Chinese version of a 10-item version of the Center for Epidemiological Studies-Depression scale [18–21] was adopted (α = 0.85). Participants were asked to rate on a 4-point scale (0 = *none*, 3 = *5–7 days*) the presence of different symptoms over the past week. Sample items are “I felt depressed” and “I felt that everything I did was an effort.” A total score of ≥12 suggests probable clinical depression in older Chinese [19,20].

#### Pain intensity

Participants were asked if they were bothered by persistent pain in the past three months, which was proposed as a yardstick for differentiating chronic from acute nonmalignant pain [22]. The 3-item pain intensity subscale of the Chronic Pain Grade questionnaire [23] was used to assess pain intensity on a 0–10 scale (α = 0.82). A sample item is “in the past three months, how intense was your worst pain rated on a 0–10 scale.” Total intensity score was obtained by averaging the items on the current, worst, and average pain in the past three months and then multiplying the mean by 10. The total has a score range of 0–100, with a higher score indicating more pain.

#### Pain self-efficacy

The 22-item Chronic Pain Self-Efficacy Scale, with subscales measuring pain management, physical function [11], and coping with pain symptoms was used. It was translated into Chinese using a translation and back-translation procedure. Items were rated from 10 to 100, with a higher score suggesting higher self-efficacy. Sample items are “How certain are you that you can make a large reduction in your pain by using methods other than taking extra medications?” and “As compared to other people with chronic medical problems like yours, how certain are you that you can manage your pain during your daily activities?” In this study, the total score, averaged across 22 items (α = 0.94), was analyzed.

#### Catastrophic cognitions

The Chinese version of the 13-item Pain Catastrophizing Scale, which measures rumination, magnification, and helplessness in three subscales, was used [24,25]. Participants were asked to reflect on past painful experiences and to indicate the extent to which they had experienced each of the 13 thoughts on a 5-point scale (0 = *not at all*, 4 = *all the time*), with a higher score indicating more serious catastrophizing thinking (α = 0.91). Sample items are “It’s terrible and I think it’s never going to get any better” and “I keep thinking about how much it [the pain] hurts”.

#### Covariates

Pain duration, pain sites, and sociodemographic information such as age, sex, and education were obtained. Self-rated health was measured by asking participants to rate their current and general health (2 items) in the past three months (0 = *poor*, 3 = *excellent*), and scores of the two items were averaged (α = 0.89). They also indicated whether they had any chronic disease among a list of 33 conditions, with an “other” category for providing information on additional conditions.

### Statistical analysis

Data were analyzed using SPSS version 21 (SPSS, Inc.; Chicago, Illinois). Alpha was set at 0.05, two-tailed. 4.7% of the sample had missing data on any variable but none had missing values in >20% of the items of any single measure. In this cases, the missing values were replaced by the person’s own mean on the remaining items of that measure. To test our theoretical model of moderated mediation, a series of linear regression analyses were performed. First, the mediating role of catastrophic cognition would be considered to exist if (a) pain intensity was associated with both catastrophizing and depressive symptoms, (b) catastrophizing was associated with depressive symptoms, and (c) the relationship between pain intensity and depressive symptoms was significantly attenuated after controlling for catastrophizing [26]. Thus, depressive symptom was regressed on pain intensity and the latter’s regression coefficient would be compared with and without catastrophizing in the model. Unbiased point estimate and 95% confidence interval of the indirect effect was calculated from 5,000 bootstrap samples using the bias-corrected bootstrapping method [27].

Second, to assess the moderating role of self-efficacy on pain intensity’s relationships to catastrophizing and depressive symptoms, two separate moderation analyses were conducted. To do this, product terms of pain intensity (centered) x self-efficacy (centered) were added to the regression models predicting catastrophizing and depressive symptoms respectively. Simple slope analyses were subsequently performed to illustrate significant interaction effects [28].

Finally, we examined whether self-efficacy moderated the mediating pathway via catastrophizing in a single model. Moderated mediation is demonstrated when the strength of an indirect effect depends on the level of the moderating variable [29]. We assessed the conditional indirect effects of pain intensity on depressive symptoms by way of catastrophizing (mediator) at specific values (-1 *SD*, mean, +1 *SD*) of pain self-efficacy (moderator). An index of moderated mediation, which is a measure of the association between an indirect effect and a moderator, was estimated, together with a 95% confidence interval, from bootstrapping 5,000 samples [30].

In all analyses, age, gender, education, self-rated health, number of chronic diseases, pain duration, number of pain sites, and pain self-efficacy were included as covariates. In models with depressive symptoms as the dependent variable, catastrophic cognition was also included as a covariate.

## Results

### Descriptive data and correlations

The mean score of pain intensity was 50.04 (*SD* = 20.04), suggesting moderate pain. In general, the participants had high self-efficacy (*M* = 70.81, *SD* = 16.02) and relatively low catastrophic thinking (*M* = 11.16, *SD* = 11.44). They had, on the average, 2.52 chronic diseases (*SD* = 1.90) but reported generally good subjective health (*M* = 1.99, *SD* = 0.58). The sample had a mean score of 7.33 (*SD* = 6.69) for depressive symptoms, with 22.1% crossing the threshold for probable clinical depression.

Table 1 shows the bivariate correlations of the variables. As expected, pain intensity was positively correlated with catastrophizing (*r* = 0.42, *p*<0.001) and depressive symptoms (*r* = 0.46, *p*<0.001). Catastrophic cognition had a highly positive correlation with depressive symptoms (*r* = 0.66, *p*<0.001), whereas self-efficacy was negatively correlated with pain intensity (*r* = -0.50, *p*<0.001), catastrophizing (*r* = -0.48, *p*<0.001), and depressive symptoms (*r* = -0.53, *p*<0.001).

**Table 1:** Descriptive statistics and product-moment correlations.

	1	2	3	4	5	6	7	8	9	10	11
1. Age	—	-0.07	-0.31	-0.10	0.03	0.03	-0.07	-0.03	0.12	-0.01	-0.26
2. Gender (female)		—	-0.20	-0.07	-0.04	0.07	0.09	0.06	0.14	0.04	-0.12
3. Education			—	0.10	0.02	-0.10	-0.05	-0.01	-0.15	0.06	0.17
4. Self-rated health				—	-0.19	-0.11	-0.18	-0.41	-0.37	-0.30	0.42
5. Number of chronic diseases					—	0.12	0.25	0.22	0.17	0.17	-0.26
6. Pain duration (years)						—	0.22	0.11	0.15	0.02	-0.09
7. Number of pain sites							—	0.28	0.27	0.21	-0.23
8. Depressive symptoms								—	0.45	0.66	-0.53
9. Pain intensity									—	0.42	-0.50
10. Pain catastrophizing										—	-0.48
11. Pain Self-efficacy											—
*M*	74.79	—	2.04	1.99	2.52	10.10	2.31	7.33	50.04	11.16	70.81
*SD*	7.20	—	0.86	0.58	1.90	10.46	1.29	6.69	20.04	11.44	16.02
%	—	85.1	—	—	—	—		—	—	—	—

*Note*. *rs* ≥ |0.09| were significant at the 0.05 level.

—not applicable.

### Pain intensity and depressive symptoms, with catastrophic cognition as mediator

Model 1 (Table 2) shows the regression results with catastrophic cognition as the dependent variable. Pain intensity was significantly associated with catastrophizing, after controlling for the covariates. Models 3 and 4 (Table 3) display the results of regression analyses showing pain catastrophizing to be the strongest predictor of depressive symptoms. Pain intensity remained significantly associated with depressive symptoms, whether catastrophic cognition, a significant predictor of depressive symptoms, was included in the model or not. However, the strength of this relationship was noticeably reduced after entering catastrophic cognition into the equation, as seen from the changes in the standardized regression coefficient of pain intensity from Model 3 (β = 0.20, *p*<0.001) to Model 4 (β = 0.09, *p* = 0.006). Thus, the condition for catastrophizing as a mediator of the relationship between pain intensity and depressive symptoms was established. Results of the bootstrap analysis showed that pain intensity’s indirect effect on depressive symptoms via catastrophizing was significant (B = 0.036, 95% CI = 0.024, 0.051).

**Table 2:** Regression of catastrophic cognition on pain intensity, self-efficacy, and covariates.

	Model 1			Model 2		
	B (*SE*)	β	*p*	B (*SE*)	β	*p*
Age	-0.16 (0.06)	-0.10	0.004	-0.16 (0.06)	-0.10	0.005
Gender (female)	-0.62 (1.08)	-0.02	0.569	-0.63 (1.07)	-0.02	0.557
Education	1.74 (0.49)	0.13	<0.001	1.66 (0.48)	0.12	<0.001
Self-rated health	-1.60 (0.72)	-0.08	0.026	-1.58 (0.71)	-0.08	0.027
Number of chronic diseases	0.11 (0.21)	0.02	0.595	0.17 (0.21)	0.03	0.414
Pain duration	-0.06 (0.04)	-0.06	0.086	-0.07 (0.04)	-0.07	0.045
Number of pain sites	0.47 (0.31)	0.05	0.132	0.55 (0.31)	0.06	0.081
Pain self-efficacy	-0.26 (0.03)	-0.37	<0.001	-0.25 (0.03)	-0.35	<0.001
Pain intensity	0.13 (0.02)	0.23	<0.001	0.13 (0.02)	0.23	<0.001
Pain intensity x Self-efficacy	†			-0.004 (0.001)	-0.12	<0.001
*R*^2^	0.320			0.333		

† not entered.

**Table 3:** Regression of depressive symptoms on pain intensity, catastrophic cognition, and covariates.

	Model 3			Model 4			Model 5		
	B (*SE*)	β	*p*	B (*SE*)	β	*p*	B (*SE*)	β	*p*
Age	-0.14 (0.03)	-0.15	<0.001	-0.09 (0.03)	-0.10	<0.001	-0.09 (0.03)	-0.10	<0.001
Gender (female)	-0.72 (0.60)	-0.04	0.229	-0.55 (0.52)	-0.03	0.290	-0.56 (0.52)	-0.03	0.278
Education	0.44 (0.27)	0.05	0.101	-0.03 (0.23)	-0.004	0.884	-0.05 (0.23)	-0.01	0.832
Self-rated health	-2.11 (0.40)	-0.18	<0.001	-1.67 (0.35)	-0.14	<0.001	-1.67 (0.34)	-0.15	<0.001
Number of chronic diseases	0.11 (0.11)	0.03	0.342	0.08 (0.10)	0.02	0.430	0.10 (0.10)	0.03	0.306
Pain duration	0.01 (0.02)	0.02	0.551	0.03 (0.02)	0.05	0.097	0.02 (0.02)	0.04	0.157
Number of pain sites	0.44 (0.17)	0.09	0.011	0.31 (0.15)	0.06	0.038	0.34 (0.15)	0.07	0.022
Pain self-efficacy	-0.16 (0.02)	-0.38	<0.001	-0.09 (0.01)	-0.21	<0.001	-0.08 (0.01)	-0.20	<0.001
Pain intensity	0.07 (0.01)	0.20	<0.001	0.03 (0.01)	0.09	0.006	0.03 (0.01)	0.09	0.004
Pain catastrophizing	†			0.27 (0.02)	0.47	<0.001	0.27 (0.02)	0.45	<0.001
Pain intensity x Self-efficacy	†			†			-0.001 (0.001)	-0.08	0.004
*R*^2^	0.399			0.547			0.552		

† not entered.

### The moderating role of self-efficacy

**Fig 2:**
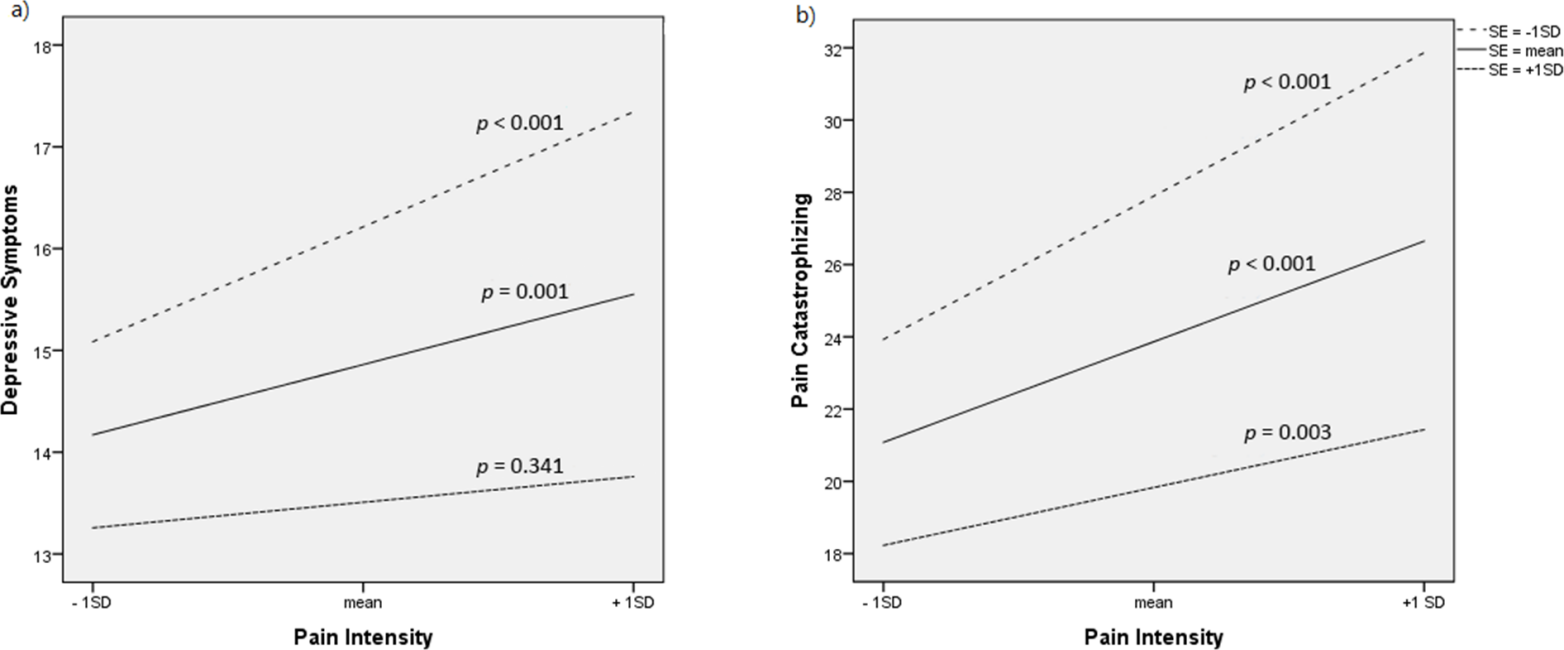
**Relationships between pain intensity and (a) depressive symptoms and (b) pain catastrophizing at -1 *SD*, mean, and +1 *SD* of pain self-efficacy**. SE = pain self-efficacy.

First, we examined whether self-efficacy moderated the association between pain intensity and depressive symptoms. The product term pain intensity x self-efficacy was significant, adding 0.5% of explained variance to the model (see Model 5, Table 3). The simple slopes at -1 *SD*, mean, and +1 *SD* of self-efficacy, shown in Fig 2A, suggested that the association between pain intensity had a strong relationship with depressive symptoms only when self-efficacy was low. When self-efficacy was high (at +1 *SD*), this relationship became nonsignificant.

Next, we tested whether self-efficacy moderated the relationship between pain intensity and catastrophic cognition. Model 2 in Table 2 showed that the interaction effect of pain intensity x self-efficacy was significant, accounting for a further 0.8% of the variance in catastrophic cognition. The simple slopes at -1 *SD*, mean, and +1 *SD* of self-efficacy are plotted in Fig 2B, which showed, again, that the strength of the relationship between pain intensity and catastrophizing was reduced with increasing levels of self-efficacy. These results suggested that the indirect effect of pain intensity on depressive symptoms, by way of catastrophizing, might depend on the level of pain self-efficacy. We then estimated the index of moderated mediation using Hayes’ algorithm [30]. This procedure yielded an estimate of B = -0.0010 (95% CI = -0.0016, -0.0004) for the moderated mediation effect. The conditional indirect effects at -1 *SD*, mean, and +1 *SD* of self-efficacy were 0.052 (95% CI = 0.033, 0.072), 0.035 (95% CI = 0.024, 0.050), and 0.019 (95% CI = 0.008, 0.033) respectively. Thus, the indirect effect of pain intensity on depressive symptoms via catastrophizing was lower in individuals with higher self-efficacy.

## Discussion

The main purpose of the study was an empirical examination of a moderated mediation model that integrates several major factors known to aggravate or alleviate depressive symptoms in those with chronic pain. To this end, a moderated mediation model was articulated and tested in a sample of Hong Kong Chinese older adults. In the cognitive theory of depression [31], catastrophic cognition is thought to be a mental set that exaggerates the threat of potentially stressful stimuli. Consistent with this view, our model assumes pain catastrophizing to be activated by the experience of bodily pain and to act as a proximal factor for depressive symptoms. Other researchers have found support for this mediating role for catastrophic cognitions [8,9]. Our study corroborates their results and found that pain intensity was associated with increased depressive symptoms through more catastrophic cognitions. However, different from previous studies which reported full mediation [8,9], we only found partial mediation. The direct effect of pain intensity on depressive symptoms in our study suggests that chronic pain itself appears to be a sufficient condition for the development of depression in older adults. More studies are needed to determine whether there remains a direct effect of pain intensity on depressive symptoms, after controlling for catastrophic cognitions.

Our results further suggest that not only does catastrophizing mediate the relationship between pain intensity and depressive symptoms, but that this mediation pathway depends on the level of pain self-efficacy. Self-efficacy, the belief that one can deal with the pain symptoms and to reduce their impact on everyday life, was hypothesized to attenuate the link between pain intensity and catastrophic cognitions. Consistent with our hypothesis, we found that self-efficacy moderated the relationship between pain intensity and catastrophic cognitions such that the relationship was weaker when self-efficacy was higher. To the best of our knowledge, this is the first study to demonstrate such a moderation effect of self-efficacy in the literature, with significant implications for theoretical development.

The relationship between self-efficacy and catastrophizing has not been clearly spelled out in the literature. Typically they are considered positive and negative cognition respectively in dealing with pain and are shown to be independently related to pain outcomes [32,33]. Such a view ignores the decades of research on the psychology of resilience and self-efficacy as a resource that contributes to resilience. Resilience refers generally to the ability to bounce back from adversity and researchers look for protective factors that attenuate the connection between adversity and negative outcomes, and self-efficacy is one such protective factor that has received consistent support in the literature [34]. In the context of coping with pain, for example, those with a high sense of self-efficacy would be more likely to view pain-related issues as challenges to be overcome, to recoup after defeats, and to keep finding ways to contain its impact [11], and hence such individuals would be less likely than those with low self-efficacy to engage in rumination and to feel overwhelmed by the pain sensation.

Resilience is usually demonstrated by a significant interaction between the adversity and the protective factor [35]. Indeed, we found that pain self-efficacy moderated the relationship between pain intensity and catastrophic cognitions as shown by a significant pain intensity x self-efficacy term. The simple slopes plotted in Fig 2B showed that the strength of the association between pain intensity and catastrophizing depended on the level of self-efficacy—this relationship was strongest when self-efficacy was low and gradually diminished with higher self-efficacy. In other words, older chronic pain patients with higher self-efficacy were less likely to engage in catastrophic cognitions than those who had a comparable level of pain but a lower sense of self-efficacy. On top of the mediational role of catastrophic cognitions, our findings suggested that the indirect effect of pain intensity on depressive symptoms, by way of catastrophizing, was moderated by pain self-efficacy as well. With increasing levels of pain self-efficacy, this indirect effect became weaker in a dose-dependent manner.

Furthermore, we found that self-efficacy also moderated the direct relationship between pain intensity and depressive symptoms. In fact, consistent with the pain literature, we found a significant association between self-efficacy and depressive symptoms; older chronic pain patients with higher self-efficacy reported less depressive symptoms than those with lower self-efficacy. However, this direct effect did not account for the entirety of the negative association between pain self-efficacy and depressive symptoms; such a negative relationship was also due to the fact that self-efficacy weakened the connection between pain intensity and depressive symptoms. This moderation effect was also reported by Pjanic and colleagues earlier [16]. Again, there was a strong relationship between pain intensity and depressive symptoms only when self-efficacy was low. At high levels of self-efficacy, older adults’ depressive symptoms was independent of the level of pain experienced (i.e., remaining relatively low even when pain was severe). The actual connection between pain self-efficacy and depressive symptoms appears to be more complicated than was assumed previously; while older chronic pain patients with higher self-efficacy are in general less depressed than those with lower self-efficacy (i.e., the direct effect), they are also less likely to feel depressed under moderate to severe pain because of the attenuated effect of pain intensity on depressive symptoms, either directly or indirectly through reduced catastrophizing.

Although catastrophic cognition was most strongly associated with depressive symptoms, the present findings suggest that pain self-efficacy should also be a key target for change in intervention programs. Along this line, Turner and colleagues found, in a randomized controlled trial, that self-efficacy uniquely mediated the effect of cognitive-behavioral treatment on pain intensity, activity interference, and jaw use limitations after one year in a group of chronic facial pain patients [36]. It is also possible that pain self-efficacy is a “common denominator” among interventions that work in the sense that effective interventions, whether adopting the mindfulness or the cognitive-behavioral approach, would promote a sense of personal efficacy in coping with pain and in reducing the impact of pain on everyday life [37].

Five limitations of this study need to be mentioned. First, the sample were only moderately depressed and did not reportedly engage in much catastrophic thinking. It would be important to attempt to replicate the present findings in a sample with more catastrophic thinking and more serious depressive symptoms. Such a sample may be more likely found in clinical settings than in the community. Second, although this study adds to the relatively small literature focusing on chronic pain in older adults, future studies should recruit a general adult sample to see if the current findings can be generalized to younger adults with chronic pain. Third, the sample was predominantly women. Although it is not uncommon to have more women than men in older samples, future research should recruit a more sex-balanced sample. Fourth, although studies of healthy individuals have found the effect of self-efficacy to be stronger in individualistic than in collectivistic cultures [38,39] similar cross-cultural comparison has not been conducted for pain patients. Thus, it is important to conduct further research to see whether the moderational roles of self-efficacy are generalizable across cultures and whether its effects are stronger in some cultural groups than others.

Finally, the cross-sectional design is limited in two ways. It has been known that estimates of mediation effects in cross-sectional models would differ from those in longitudinal models, due to the inability of cross-sectional data to model changes in the mediator and the dependent variable over time [40]. The current results about the mediational role of catastrophizing should therefore be taken with caution, and corroboration through analyzing a 3-wave longitudinal model is needed in future research to strengthen the support for this theoretical model. Moreover, the causal directions are not necessarily one way from pain intensity to catastrophizing and to depressive symptoms [41,42]. Although the possibility of reverse causation does not in itself invalidate our theoretical model, the true relationships among these factors may be a lot more complex that implies a downward spiral in pain and depression over time, unless one develops a sense of efficacy in dealing with pain-related issues. Future research should aim at a longitudinal design with several waves with carefully spaced-out intervals in order to test moderated mediation and reverse causation simultaneously.

## Conclusions

This is the first study to demonstrate in a moderated mediation model concerning the protective role of pain self-efficacy in the process leading to pain-related depressive symptoms. On top of the direct effect of self-efficacy on depressive symptoms, it is important to note the moderational role that self-efficacy plays in this process. As a protective factor, pain self-efficacy attenuates the direct relationship between chronic pain and depressive symptoms and also their indirect relationship through reducing catastrophizing about pain. The study provides a new perspective to understand the complex relationships among pain intensity, catastrophizing, and self-efficacy in accounting for depressive symptoms in older chronic pain patients. Our study suggests the potential need of considering both pain self-efficacy and catastrophizing in the design of pain intervention programs.
